# Supplementary material for: New Generations of Tyrosine Kinase Inhibitors in Treating NSCLC with Oncogene Addiction: Strengths and Limitations
Source: Cancers (Basel). 2023 Oct 20;15(20):5079. doi: 10.3390/cancers15205079 (PMC10605462; doi:10.3390/cancers15205079)
Supplement: Supplementary file 1 [file cancers-15-05079-s001.zip › cancers-2665418-supplementary.pdf]

Supplementary Materials: New Generations of Tyrosine Kinase Inhibitors in Treating NSCLC with Oncogene Addiction: Strengths and Limitations

Ilaria Attili, Carla Corvaja, Gianluca Spitaleri, Ester Del Signore, Pamela Trillo Aliaga, Antonio Passaro and Filippo de Marinis

Table S1. 4th generation EGFR TKIs properties compared to those of osimertinib.

Drug	IC_{50}	CNS Penetrant	Clinical trial
Osimertinib	EGFR 19Del/C797S = 304.39 nM<br>EGFR 19Del/T790M/C797S =124.82nM<br>EGFR L858R/C797S = 573.72 nM	Y	FLAURA
EAI045	EGFRC7697S = only when added cetuximab	-	Withdrawn
JBJ-04-125-02	EGFRL858R/T790M= 0.26 nmol/l	-	Withdrawn
BLU-701	EGFRex19del/C7697S = 0.5 nM<br>EGFRL858R/C797S= 1.5 nM	Y	None
BPI-361175	No data available	Y	Phase I/II trial<br>Post-3rd TKI<br>NCT05393466
QLH11811	EGFRex19del/T790M/C7697S = 2.6 nM<br>EGFRL858R/T790M/C797S= 2.6 nM	-	Phase I trial<br>Either Tx naïve or post-TKIs<br>NCT05555212
BDTX-1535	EGFRC797S < 10nM	Y	Phase I EGFR+ NSCLC* or glioblastoma<br>NCT05256290
BBT-207	EGFRex19del/T790M/C7697S <10nM<br>EGFRL858R/T790M/C797S < 10 nM	Y	Phase I/II trial<br>Post-3rd TKI<br>NCT05920135
JIN-A02	EGFRex19del/T790M/C7697S = 4.7nM<br>EGFRL858R/T790M/C797S= 12.8 nM	Y	Phase I/II trial<br>Post TKI and CT<br>NCT05394831
HS-10375	No data available	-	Phase I/II trial<br>Post-3rd TKI<br>NCT05435248
TAS3351	No data available	-	Phase I/II trial<br>Post-3rd TKI<br>NCT05765734
BLU-945	EGFRex19del/C7697S = 0.5 nM<br>EGFRL858R/T790M/C797S= 0.5 nM	-	Phase Ib/II<br>Post-3rd TKI<br>SYMPHONY<br>NCT04862780<br>(plus osimertinib)
BBT-176	EGFR 19Del/C797S = 4.36 nM<br>EGFR 19Del/T790M/C797S = 1.79 nM<br>EGFR L858R/C797S = 5.35 nM	-	Phase I/II trial<br>Post-3rd TKI<br>NCT04820023
H002	EGFREx19del/T790M/C797S < 5 nM	Y	Phase I/II

	EGFRL858R/T790M/C797S < 5 nM		Post-3rd TKI<br>NCT05519293
WJ13404	No data available	-	Phase I/II<br>**C797S+**<br>Post-3rd TKI<br>NCT05662670

Abbreviations: IC50 = Half-maximal inhibitory concentration CNS = central nervous system; Y = yes; EGFR = Epidermal Growth Factor Receptor; NSCLC = Non-Small-Cell Lung Cancer; TKI(s) = Tyrosine Kinase Inhibitor(s); CT = chemotherapy. *dose expansion catered for EGFR C797S+ NSCLC patients.

Table S2. Summary of EGFR Ex20ins TKi inhibitors.

Drug	EGFRex20ins IC_{50}	Clinical trial	Results	Ongoing CTs
Mobocertinib (TAK-788) 160 mg OD	4.3-22.5 nM	EXCLAIM<br>NCT02716116<br>Zhou 2022[1]	ORR 28%<br>mPFS 7.3 months	NCT02716116<br>(w or wout BM+)
Poziotinib (HM781-36B) 16 mg OD	1.0 nM	ZENITH20<br>[NCT03318939]<br>Le 2020[2]	ORR 14.8%<br>mPFS 4.2 months	NCT03318939<br>8 mg BID
TAS6417 (CLN-081) 100 mg BID	3-21 nM	NCT04036682<br>Yu 2022[3]	ORR 41%<br>mPFS 12 months	NCT04036682<br>Ph II part<br>BM+ and prior TKIs
Sunvozertinib (DZD9008) 400 mg OD	2.1 nM	WU-KONG1<br>[NCT03974022]<br>WU-KONG2 [CTR20192097]<br>Wang 2022[4]	ORR 37.5%<br>mPFS NR (> 4 months)	NCT05668988<br>Ph III S vs 1st line CT
Tuxobertinib (BDTX-189) 800 mg OD	10-100 nM	Masterkey-01 NCT04209465	5 pts EGFREx20ins<br>5 pts HER2Ex20ins	NCT04209465<br>Enrollment is ongoing in non-fasting QD and BID co-horts, and the low fat food effect cohort
Furmonertinib 160 mg or 240 mg OD	11-81 nM	FAVOUR<br>NCT04858958<br>Han 2021[5]	Pretreated<br>160 mg ORR 40%<br>240 mg ORR 50%<br>Tx naïve<br>ORR 80%	phase II trial NCT05466149<br>**Phase III trial**<br>**FURMO-004**<br>NCT to be determined<br>240 mg OD vs 160 mg OD<br>Vs CT<br>**Phase Ib trial**<br>**FURMO-002**<br>Dose escalation from 240 up to 320 mg OD

Abbreviations: CTs = clinical trials; OD = once daily; nM = nanomole; ORR = overall response rate; mPFS = median progression-free survival; NR = not reached; pts = patients; Tx naïve = treatment naïve; w= with; wout= without; BM = brain metastases; BID = two times a day; NCT National = Cancer Center trial ID.

Table S3. Summary of HER2ex20 ins TKIs.

Drug	HER2ex20ins IC_{50}	Clinical trial	Results	Ongoing CTs
Mobocertinib[6]	YVMA = 70nM<br>G776->VC= 10 nM<br>GSP= 10 nM	-	-	NCT02716116<br>Cohort 2 HER2Ex20ins with no BM
Poziotinib (HM781-36B)	1.9 nM	ZENITH20<br>[NCT03318939]<br>Le 2022[7]	ORR 28%<br>mPFS 5.1 months	NCT03318939<br>8 mg BID
Tarloxotinib	0.15-4.34 nM	RAIN-701<br>Phase I/II trial<br>[NCT03805841]<br>Liu 2020[8]	ORR 22%	RAIN-701<br>[NCT03805841]<br>Phase II part
Pyrotinib	G776YVMA= 180 nM*	Phase II trial<br>NCT02834936<br>Zhou 2020[9]	ORR 30%<br>mPFS 6.9 months	PEER20 trial<br>Phase II trial<br>(NCT04063462)<br>PYRAMID-1 trial<br>Phase III trial<br>(vs docetaxel)<br>(NCT04447118)<br>Phase II<br>(pyrotinib + PD1 abs)<br>(NCT04144569)
Tuxobertinib (BDTX-189)	13 nM^	Masterkey-01<br>NCT04209465	5 pts EGFREx20ins<br>5 pts HER2Ex20ins	NCT04209465<br>Enrollment is ongoing in non-fasting QD and BID cohorts, and the low fat food effect cohort
TAS2940[10]	1.54-3.28 nM	-	-	NCT04982926<br>Phase I trial

Abbreviations: CTs = clinical trials; OD = once daily; nM = nanomole; ORR = overall response rate; mPFS = median progression-free survival; NR = not reached; pts = patients; BM = brain metastases; BID = two times a day. *refers to growth inhibition of patient-derived organoids and xenografts from a 42-year-old female with HER2-A775_G776YVMA-inserted advanced lung adenocarcinoma.

Table S4. Ongoing clinical trials of RAS inhibitors.

Trial	Phase	Pt N	Drug	Disease	Key inclusion criteria	Location
CODEBREAK-IGR<br>NCT05631249	II	40	Sotorasib	KRASG12C NSCLC	Post 1st line<br>No Prior KRAS-I	France
SOLUCOM<br>NCT05311709	II	100	Sotorasib	KRASG12C NSCLC + comorbities	Post 1st line<br>60 pts ECOG PS2<br>No prior KRAS-I	Norway
ECOG-ACRIN<br>LUNG-MAP<br>NCT04625647	II	116	Sotorasib	KRASG12C NSCLC	No prior KRAS-I	USA
Breakthrough<br>NCT05451056	II	37	Sotorasib	KRASG12C NSCLC	Post 1st line tx	Korea
LungKG12Ci<br>NCT05273047	obs	300	Sotorasib	KRASG12C NSCLC	Real world data	France
MERIT-lung<br>NCT05398094	II	43	Sotorasib	KRASG12C NSCLC	Stage III Ineligible for CRT	Spain
KRYSTAL12<br>NCT04685135	III	450	Adagrasib<br>Vs<br>docetaxel	KRASG12C NSCLC	Post CT+ ICI<br>No prior RAS-I	Worldwide
ADEPPT<br>NCT05673187	II	68	Adagrasib	KRASG12C NSCLC	Elderly<br>Post CT+/-ICI<br>No prior RAS-I	Europe
KRYSTAL 21	II	200	Adagrasib	KRASG12C NSCLC	Post CT+/-ICI	USA

NCT05853575			600 vs 400 mg BID		No prior RAS-I	
NAUTIKA1 NCT04302025	II	85	Divarasib	gene addicted NSCLC	Stage Ib-IIIB Neoadjuvant setting Biomarker-driven platform	USA
KontRASt-01 NCT04699188	I/II	475	JDQ433	KRASG12C solid tumours	No prior RAS-I	Worldwide
KontRASt-02 NCT05132075	III	360	JDQ433 Vs docetaxel	KRASG12C NSCLC	Post CT+ ICI No prior RAS-I	Worldwide
STRIDER NCT05999357	II	42	JDQ433	KRASG12C NSCLC	BM+ No Prior RAS-I or at least after 1 year of interval	Nederland
KontRASt-06 NCT05445843	II	120	JDQ433	KRASG12C NSCLC	Tx naïve PD-L1 neg or PD-L1 +/SKT11+	Worldwide
Neoadjuvant Platform NCT05714891	II	27	JDQ433	KRASG12C NSCLC	Neoadjuvant setting Stage IA2 to IIIA	Canada
NCT04956640	I	400	LY3537982	KRASG12C solid tumours	Post CT +ICI	Worldwide
NCT04973163	I	29	BI 1823911	KRASG12C solid tumours	No prior RAS-I allowed	USA, Europa
NCT05002270	I/II	124	Glecirasib (JAB-21822)	KRASG12C solid tumours	Dose exp CRC drug + cetuximab	USA
NCT05737706	I/II	304	MRTX1133	KRASG12D solid tumours	Post standard tx	USA

Abbreviations: Pt N = patient number; obs = observational; vs = versus; BID = two times a day; NSCLC = non-small-cell lung cancer; KRAS-I(s) = KRAS inhibitors; ECOG PS = Eastern Cooperative Oncology Group Performance Status; Tx = treatment; CT = chemotherapy; ICI = immune-checkpoint inhibitor; PD-L1 = Programmed Cell Death Ligand 1; CRT = chemo-radiotherapy; BM = brain metastases.

Table S5. Ongoing clinical trials of RAF inhibitors.

Trial	Phase	Pt N	Drug(s)	Disease	K Inclusion criteria	Location
ENCO-BRAF NCT04526782	II	119	Encorafenib + Binimetinib	BRAFV600E	Tx naïve or post CT+/-ICI No prior BRAF/MEK-I	France
OCEANII NCT05195632	II	55	Encorafenib + Binimetinib	BRAFV600E	Tx naïve or post CT No prior BRAF/MEK-I	China
NCT05065398	II	20	HLX208 (BRAF-I)	BRAFV600E	Post CT+/-ICI No prior BRAF/MEK-I	China, Taiwan
NCT05900219	II	75	Vemurafenib+ HL-085 (MEK-I)	BRAFV600E	No prior BRAF/MEK-I	China
RAMP202 NCT04620330	II	100	avutometinib (VS-6766) (RAF/MEK clamp) + Defactinib (FAK inhibitor)	KRASG12V/other BRAFV600E/other	Cht 1/2 KRASG12V Cht 3 KRAS other Cht 4 BRAFV600E Cht 5BRAFother No prior KRAS/BRAF/MEK-I	USA, Europe
NCT04913285	I	262	Exarafenib (KIN-2787) panRAF-I+/- binimetinib	BRAF/NRAS+ solid tumours and NSCLC/Melanoma	Dose Exp No prior BRAF/MEK-I	USA, China, Taiwan, Korea, Australia, Europe
NCT05786924	I	140	BDTX-4933 panRAF	BRAF/RAS/MAPK+ solid tumours	Dose Exp CHT1 NSCLC BRAF class II or KRAS NON G12C CHT3 BRAF class I	USA
NCT05668585	I/II	122	CFT1946 (RAF-I) +/- Trametinib	BRAFV600E solid tumours	Prior or naïve RAF-I permitted	USA, France, Spain
ENHANCE NCT05275374	I/II	221	XP-102 RAF-I) +/- Trametinib	BRAFV600E solid tumours	Prior RAF/MEK-I permitted	China

NCT05501912	I	72	ABM-1310<br>RAF-I	BRAFV600E solid tumours	Prior RAF/MEK-I permitted	China, Shanghai
NCT04190628	I	112	ABM-1310<br>RAF-I<br>Cobimetinib<br>MEK-I	BRAFV600E/ or other solid tumours	Prior RAF/MEK-I permitted	USA
NCT05355701	I	174	PF-07799933 (ARRY-440)<br>RAF-I-/+ binimetinib or cetuximab	BRAF class II/III solid tumours	BRAF class II/III	USA, Canada, Israel
NCT05538130	I	124	Encorafenib +<br>PF-07799544<br>MEK-I	BRAF V600E or class II solid tumours	BRAF V600E or class II	USA, Canada
NCT04985604	I/II	168	Tovorafenib<br>PanRAF-I+<br>Pimasertib<br>MEK1-2-I	MAPK Pathway Aberrations<br>Solid tumours	Pts must have<br>BRAF fusion, CRAF/RAF1 fusion, or CRAF/RAF1 amplification<br>No Prior RAF/MEK-I	USA, Australia, Europe, Korea
NCT04892017	I/II	323	DCC-3116+/-<br>Trametinib, bimetinib or sotorasib	MAPK Pathway Aberrations<br>Solid tumours	RAS, NF1, or RAF mutations<br>No Prior RAS/RAF/MEK-I if discontinued due to safety	USA
NCT04800822	I	196	PF-07284892<br>SHP2-I +<br>binimetinib	BRAF class III solid tumours	A/B ALK/ROS1 lorlatinib<br>C /D CRC BRAF<br>E BRAF Solid tumours	USA

Abbreviations: Pt N = patient number; NSCLC = non-small-cell lung cancer; (B)RAF/MEK/KRAS/NF1-I(s) = KRAS inhibitors; ECOG PS = Eastern Cooperative Oncology Group Performance Status; Tx = treatment; CT = chemotherapy; ICI = immune-checkpoint inhibitor; Cht = cohort; Dose Exp= dose expansion. CFT1946 is an orally active and selective target ligand for BRAF kinase. CFT1946 is a degrader of mutant BRAFV600E, G469A, G466V and p61-BRAFV600E.

Table S6. Ongoing clinical trials of novel MET-inhibitors in MET dysregulated NSCLC or solid tumours.

Trial	Phase	Pt N	Drug	Disease	Key inclusion criteria	Location
SPARTA<br>NCT03175224	I/II	344	Bozitinib<br>(APL-101 or PLB1001)	MET+ solid tumours or NSCLC	Solid tumors or NSCLC harbouring MET alterations<br>MET-I naïve or refractory	Worldwide
KUNPENG<br>NCT04258033	II	185	Bozitinib<br>(APL-101 or PLB1001)	MET+	Must have c-Met dysregulation adv NSCLC<br>Not mentioned Prior MET-Is in the criteria	China
NCT04992858	II	80	Ningetinib	METex14 NSCLC	METex14 in lx or tx<br>Prior therapies with MET-Is are not allowed	China (not yet recruiting)
SHIELD-1<br>NCT03993873	I/II	180	TPX-0022<br>(elzovantinib)	MET+ NSCLC, gastric or solid tumours	METex14 or MET AMP<br>No restrictions on prior MET-Is	USA, Korea, France, Spain<br>**Enrollment completed**
NCT04270591	I/II	183	Glumetinib (SCC244)	MET+/METex14 NSCLC	Cohort 1 (China):<br>MET OE, MET AMP, METex14<br>Cohort 2 (USA) METex14<br>Only in phase I Prior MET-Is allowed	China, Usa
NCT02929290	Ib	80	BPI-9016M, a dual MET/Axl inhibitor	METex14 or MET OE NSCLC	MET OE or METex14<br>Adv NSCLC<br>Prior type I MET-Is are allowed	China
NCT04052971	I/II	78	ABN401 (selective MET-I)	MET+ NSCLC or solid tumours	Phase I MET+<br>Phase II METex14	Korea, Australia

					Only in phase I Prior MET-Is allowed	
NCT05752552	I	15	DO-2 (selective MET-I)	MET+ solid tumours	MET mut or MET AMP (≥10 copies)<br>No restrictions on prior MET-Is	Belgium, Netherlands

Abbreviations = pt N = patient number; lx = liquid biopsy; tx = tissue biopsy; MET OE = MET overexpression; METex14 = MET exon 14 skipping mutations; MET AMP = MET amplification; MET-I(s) = MET inhibitor(s); NSCLC = Non-small-cell lung cancer. .

Table S7. Ongoing clinical trials of novel ALK TKIs.

Trial	Phase	Pt N	Drug	Disease	Key inclusion criteria	Location
NCT03607188	I	18	Alkotinib<br>(2nd ALK-I)	ALK+ NSCLC	Post-crizotinib	Shanghai
NCT04211922	II	104	Alkotinib<br>2nd ALK-I	ALK+ NSCLC	Post-crizotinib	Shanghai
NCT05441956	I	100	TGRX-326<br>(3rd ALK-I)	ALK+ or ROS1+ NSCLC	Post 1st/ 2nd ALK-I	China
NCT04237805	I/II	280	Foritinib<br>(SAF-189s)<br>(3rd ALK-I/ROS-1)	ALK+ NSCLC	Tx naïve and Refractory	Shanghai
NCT05257512	I/II	70	SY-3505<br>(3rd ALK-I)	ALK+ NSCLC	Refractory	China
NCT05055232	I	120	XZP-3621<br>(3rd ALK-I)	ALK+ NSCLC	Naïve/refractory	China
NCT05482087	II	190	XZP-3621<br>(3rd ALK-I)	ALK+/ROS1+ NSCLC	Naïve/refractory	China
NCT05204628	RPIII	238	XZP-3621<br>Vs<br>crizotinib	ALK+ NSCLC	Tx Naive	China
NCT03917043	I	150	APG-2449<br>FAK/ALK/ROS1 I	Solid tumours	Expansion phase<br>Tx naïve or pretreated ALK or ROS1+ NSCLC	China
NCT04849273	I	11	TPX-0131<br>4th ALK-I	ALK+ NSCLC or solid tumours	Pretreated with up 3 ALK-Is	USA, Korea, Australia<br>Terminated (Adverse change in the risk/benefit)
ALKOVE-1<br>NCT05384626	I/II	214	NVL-655<br>4th ALK-I	ALK+ NSCLC or solid tumours	Expansion Phase<br>Cohorts 2 A-C NSCLC<br>Cohorts 2D other solid tumours	USA, Europe

Abbreviations: Pt N = patient number; ALK = Anaplastic Lymphoma Kinase; ALK-I(s) = ALK inhibitor(s); Tx = treatment; NSCLC = Non-small-cell-lung cancer.

Table S8. Ongoing clinical trials of novel ROS1 TKIs.

Trial	Phase	Pt N	Drug	Disease	Key inclusion criteria	Location
NCT04603807	III	220	Crizotinib<br>Vs<br>entrectinib	ROS1+ NSCLC	Tx naïve<br>With or without BM	Worldwide
BFAST<br>NCT03178552	II	1000	entrectinib	ROS1+ NSCLC	Tx naïve<br>ctDNA+ for ROS1+	Worldwide
NCT05170204	III	320	Entrectinib<br>Vs durvalumab	ROS1+NSCLC	Stage III NSCLC<br>A1 ALK+ Alectinib<br>A2 ROS1+<br>A3 RET+	Worldwide
TRIDENT-1<br>NCT03093116	I/II	500	repotrectinib	ALK, ROS1, or NTRK solid tumours	EXP-1: ROS1 TKI-naïve ROS1+ NSCLC	Worldwide

					EXP-2: 1 Prior ROS1 TKI and 1 Platinum based CT ROS1+ NSCLC<br>EXP-3: 2 Prior ROS1 TKIs ROS1+ NSCLC (No CT or ICI)<br>EXP-4: 1 Prior ROS1 TKI ROS1+ NSCLC (No CT or ICI)<br>EXP-5: TRK TKI-naïve NTRK+ solid tumors<br>EXP-6: TRK TKI-pretreated NTRK+ solid tumors	
NCT04395677	II	106	taletrectinib	ROS1+ NSCLC	ROS1-I naïve or post-crizotinib	China
TRUST II NCT04919811	II	154	taletrectinib	ROS1+ NSCLC	TKI tx naïve or treated with prior ROS1-Is	Worldwide
ARROS-1 NCT05118789	I/II	359	NVL-520	ROS1+ solid tumours or NSCLC	2a: ROS1 naïve/ up 1L CT<br>2b: 1L ROS1-I/ CT naïve<br>2c: 1L ROS1-I/ 1L CT<br>2d: ≥2L ROS-Is/1L CT<br>2e: solid tumours	Worldwide
NCT05765877	II	26	Iruplinalkib WX-0593	ALK or ROS1+ NSCLC	Stage IB-IIIA	China
NCT05351320	II	40	Iruplinalkib WX-0593 plus CRT	ALK or ROS1+ NSCLC	Stage III	China
NCT04197076	II	200	TKIs	NSCLC	IIB-IIIB stage<br>A: PD-L1 ≥ 50% ICI<br>B: EGFR/ALK or ROS1+<br>C: non-gene addicted	China
NCT03607188	I	18	Alkotinib	ALK or ROS1+ NSCLC	Pretreated with CT or crito-tinib	China
NCT05441956	I	100	TGRX-326	ALK+ or ROS1+ NSCLC	Post 1st/ 2nd TKIs	China
NCT04237805	I/II	280	Foritinib (SAF-189s)	ALK+ or ROS1+ NSCLC	Tx naïve and Refractory	Shanghai
NCT04996121	I/II	360	XZP-5955	NTRK+ or ROS1+ solid tumours	Dose exp<br>NTRK solid tumours<br>Tx naïve or post crizotinib<br>ROS1+ NSCLC	China
NCT03917043	I	150	APG-2449 FAK/ALK/ROS1 I	Solid tumours	Expansion phase<br>Tx naïve or pretreated ALK or ROS1+ NSCLC	China
ALBATROS NCT04621188	II	84	lorlatinib	ROS1+ NSCLC	Post-crizotinib or entrectinib<br>Post-CT	France
NCT03612154	II	35	lorlatinib	ROS1+ NSCLC	Tx naïve or post CT<br>**No prior ROS1-I**	Korea
NCT01639508	II	86	cabozantinib	ROS1+ NSCLC	Group A RET+<br>Group B NTRK, AXL or MET<br>Group C ROS1<br>Group D RET+ prior RET-I	USA

Abbreviations: Pt N= patient number; ROS1 = c-ros oncogene 1; ROS1-I(s) = ROS1 inhibitors ; Tx = treatment; NSCLC = Non-small-cell-lung cancer; BM = brain metastases; TKI(s) = tyrosine kinase inhibitor(s); TX = treatment; EXP = expansion; CT= chemotherapy; ICI = immunocheckpoint inhibitor.

Table S9. Ongoing clinical trials of novel NTRK-inhibitors.

Trial	Phase	Pt N	Drug	Disease	Key inclusion criteria	Location
NAVIGATE NCT02576431	II	204	Larotrectinib	NTRK+ solid tumours	1: NSCLC<br>2: thyroid carcinoma<br>3: soft-tissue sarcoma<br>4: colorectal cancer<br>5 Salivary tumours<br>6: biliary tumours<br>7: Primary CNS<br>8: other solid tumours<br>9: solid tumours without confirmed NTRK gene fusions<br>10:melanoma/non secretary breast cancer/colorectal cancer<br>11: all tumour types without measurable disease	Worldwide
NCT05192642	Obs.	368	larotrectinib	NTRK solid tumours	Real world data of larotrectinib outside clinical trials	USA
LAROTRACKING NCT04814667	Obs	27	larotrectinib	NTRK solid tumours	Real world data of larotrectinib	France
ON-TRK NCT04142437	Obs	300	larotrectinib	NTRK solid tumours	Real world data of larotrectinib	worldwide
NCT04945330	Obs	100	larotrectinib	NTRK solid tumours	Real world data of larotrectinib	Japan
NCT04879121	II	13	larotrectinib	NTRK AMP solid tumours	NTRK amplified<br>No prior treated with NTRK-Is	USA
TRUMP NCT03574402	II	400	ensartinib	ROS1+ or NTRK+ NSCLC	Tx naïve<br>Arm 5 ROS1+<br>Arm 6 NTRK+	China
TRIDENT-1 NCT03093116	I/II	500	repotrectinib	ALK, ROS1, or NTRK solid tumours	EXP-1: ROS1 TKI-naïve ROS1+ NSCLC<br>EXP-2: 1 Prior ROS1 TKI and 1 Platinum based CT ROS1+ NSCLC<br>EXP-3: 2 Prior ROS1 TKIs ROS1+ NSCLC (No CT or ICI)<br>EXP-4: 1 Prior ROS1 TKI ROS1+ NSCLC (No CT or ICI)<br>EXP-5: TRK TKI-naïve **NTRK+ solid tumors**<br>EXP-6: TRK TKI-pretreated NTRK+ solid tumors	Worldwide
NCT03556228	I	74	VMD-928	NTRK1+ solid tumours or lymphoma	NTRK1+ solid tumours or lymphoma progressing on larotrectinib or entrectinib	USA
NCT04996121	I/II	360	XZP-5955	NTRK+ or ROS1+ solid tumours	Dose exp<br>NTRK solid tumours<br>Tx naïve or post crizotinib<br>ROS1+ NSCLC	China

Abbreviations: Pt N= patient number; ROS1 = c-ros oncogene 1; NTRK = Neurotrophic tyrosine receptor kinase; NTRK-I(s)= NTRK inhibitor(s); ROS1-I(s) = ROS1 inhibitors ; Tx = treatment; NSCLC = Non-small-cell-lung cancer; BM = brain metastases; TKI(s) = tyrosine kinase inhibitor(s); TX = treatment; EXP = expansion; CT= chemotherapy; ICI = immunocheckpoint inhibitor.

Table S10. Ongoing clinical trials of novel RET TKIs.

Trial	Phase	Pt N	Drug	Disease	Key inclusion criteria	Location
LIBRETTO-001 NCT03157128	I/II	875	Selpercatinib	RET+ solid tumours	Tx naïve or pretreated RET+ solid tumours **Multitargeted inh allowed**	Worldwide
LIBRETTO-432 NCT04819100	III	170	Selpercatinib after surgery or RT	RET+ NSCLC	IB-IIIA RET Fusion-Positive NSCLC Following definitive locoregional tx	Worldwide
LIBRETTO-431 NCT04194944	III	250	Selpercatinib Vs CT +/-ICI	RET+NSCLC	Tx naïve RET fusion+ on tissue or lx	Worldwide
NCT05170204	III	320	Pralsetinib Vs durvalumab	Stage III NSCLC	Stage III NSCLC After CRT Cohort A3 RET+	Worldwide
acceleRET NCT04222972	III	226	Pralsetinib Vs CT	RET+ NSCLC	**Tx naïve RET fusion+ on tissue**	worldwide
NCT01639508	II	86	cabozantinib	RET+, ROS1+ or NTRK+ NSCLC	**A: RET+** B: NTRK+, MET+ or AXL+ C ROS1+ **D RET+ prior treated RET-Is**	USA
NCT05117658	II	83	HA121-28	RET+ NSCLC	After at least one CT line **No prior RET-Is**	China
MARGARET NCT04683250	I/II	202	TAS0953/HM06	RET+ solid tumours	RET+ solid tumours Progressing on standard tx **Prior RET-Is allowed**	USA, Japan
NCT05278364	I/II	184	SY-5007	RET+ solid tumours	RET+ solid tumours Progressing on standard tx No prior RET-Is	China
NCT06031558	III	120	SY-5007	RET+ NSCLC	Tx naïve RET fusion on tx or lx	Shanghai
NCT05675605	I/II	248	TY-1091	RET+ solid tumours	RET fusions solid tumours	China
NCT05451602	I/II	456	HEC169096	RET+ solid tumours	RET fusions solid tumours No restrictions on prior tx lines	China

Abbreviations: Pt N= patient number; lx = liquid biopsy; inh = inhibitor; RET = rearranged during transfection; ROS1 = c-ros oncogene 1; NTRK = Neurotrophic tyrosine receptor kinase; RET-(S) = RET inhibitors; NTRK-I(s)= NTRK inhibitor(s); ROS1-I(s) = ROS1 inhibitors ; Tx = treatment; NSCLC = Non-small-cell-lung cancer; BM = brain metastases; TKI(s) = tyrosine kinase inhibitor(s); TX = treatment; EXP = expansion; CT= chemotherapy; ICI = immunocheckpoint inhibitor.

Surufatinib inhibits VEGFR1/2/3, FGFR1, and CSF-1R; **Sarilumab** is an IL6-receptor Antibody; **Dalpiciclib** (CDK4/6 inhibitor) already approved in China for breast cancer; **pelcitoclax (APG-1252)** is an anti-Bcl2; **pamiparib** is an investigational Poly (ADP-ribose) polymerase (PARP) inhibitor recently approved in China for the treatment of germline BRCA mutation-associated recurrent advanced ovarian, fallopian tube or primary peritoneal cancer previously treated with two or more lines of chemotherapy; **VIC-1911** is an Aurora A inhibitor; **Minnelide** is an oral anti-super-enhancer drug that inhibits MYC expression; **Telaglenastat** (CB-839) (glutaminase inhibitor)**envafolimab** is anti-PDL1 agent; LP300 is a TRX and GRX inhibitor; **patritumumab-deruxtecan** a new ADC anti-HER3 conjugated to deruxtecan; **Quaratusugene ozeplasmid** consists of non-viral lipid nanoparticles that encapsulate a DNA plasmid with the TUSC2 tumor suppressor gene; **PM8002** is a bifunctional antibody antiPDL1/VEGF

Table S11. Ongoing trials of TKI-based combinations in *EGFR* or *HER*2 Exon 20 insertions.

Trial	Phase	Pt N	Drugs	Disease	Key inclusion criteria	Location
CONCERTO NCT05241873	I/II	322	BLU-451+/-CT	EGFREx20ins	Several CTs<br>All EGFREx20ins<br>A CT ami or mobo<br>B Post CT<br>C BM<br>D prior CT Ami and mobo<br>E Tx naive<br>F/G Uncommon	USA/Canada/Japan Taiwan
NCT04974879	II	20	Osimertinib + bevacizumab	EGFREx20ins	Not indicated prior Tx	China
NCT05132777	II	155	Osimertinib + JMT101 (ab antiEGFR)	EGFREx20ins	after CT	Shanghai
NCT05751187	II	54	Pembrolizumab Plus Bevacizumab Plus CT	EGFREx20ins	Tx naive	Shanghai
NCT04144569	II	30	Pyrotinib + PD1	HER2Ex20ins	Post-CT	China
NCT05745740	I/II	26	Pyrotinib + Disitamab Vedotin (RC48-ADC)	HER2Ex20ins	Post-CT and ICI	Shanghai
NCT05016544	I/II	48	Pyrotinib + inetetamab (anti-HER2)	HER2 mut or AMP	Post-CT<br>No prior HER2-Is	China
NCT05482568	I/II	324	Pyrotinib + SHR-A1811+ SHR-1316 Anti-PDL1	HER2	Post CT<br>Not specified HER2 alterations needed	China
NCT05847764	II	95	Disitamab Vedotin + Furmonertinib or CT	HER2 alterations	Arm 1/2 HER2 alterations w or wout EGFR+ tx naïve<br>Arm 3 pretreated HER2 mutated	China
NCT04324125	Obs	70	CT+/-ICI	HER2ins or AMP	Tx naive	China
NCT05681780	I/II	20	TILs +Nivolumab+ CTX+IL2	EGFR, ALK, ROS1, or ERBB2 alterations	Post CT	USA<br>Florida
NCT04042701	Ib	115	T dxt + pembro	HER2 OE or mutated NSCLC or Breast	Post standard tx	USA, France, UK, Spain
COMBI-TED NCT04884282	II	105	Tedopi + Nivo<br>Tedopi + TXT<br>TXT	NSCLC wout EGFR/ ALK/ROS1	Tedopi vaccine against 5 antigenes CEA, p53, HER2, MAGE-2/3	Italy
NCT04278144	I/II	390	BDC-1001 (antiHER2 stimulating Ab) -/+ nivolumab	HER2 OE or AMP solitd tumours	Post CT	USA, Korea, Spain
NCT04143711	I/II	490	DF1001 (target NK and Tcell)+ nivo<br>Or PTX	Solid tumours	Dose esc HER2 OE AMP or mut<br>Dose exp NSCLC HER2 low/high chts	USA, Europe

Abbreviations: Pt N = patient number; EGFR = Epidermal Growth Factor Receptor; HER2 = human epidermal growth factor receptor 2; Ami = amivantamab; mobo = mobocertinib; wout = without; Tx

= treatment; CT = chemotherapy; TKI(s) = Tyrosine kinase inhibitor (s); NSCLC = Non-small-cell lung cancer.

Table S12. Ongoing clinical trials of KRAS inhibitor-based combinations.

Trial	Phase	Pt N	Drugs	Disease	Key inclusion criteria	Location
CodebreaK202 NCT05920356	III	750	Sotorasib+ CT Vs Pembro + CT	KRASG12G NSCLC	Tx naïve PDL-1 negative	Worldwide
NCT05118854	II	27	Sotorasib + CT	KRASG12C NSCLC	Stage IIA-IIIB	USA
NCT05180422	II	43	Sotorasib + MVASI	KRASG12C NSCLC	BM+ No prior KRAS-I	USA
NCT05054725	II	47	Sotorasib+ RMC-4630 (SHP2-inh)	KRASG12C NSCLC	No prior KRAS-I	Worldwide
RAMP203 NCT05074810	Ib/II	53	Sotorasib + Avutometinib (VS-6766) (MEK-I)	KRASG12C NSCLC	Part 1 No prior KRAS-I Part 2 Prior KRAS-I	USA
NCT04892017	I/II	323	DCC-3116+/- Trametinib, bimetinib or sotorasib	MAPK Pathway Aberrations Solid tumours	RAS, NF1, or RAF mutations No Prior RAS/RAF/MEK-I	USA
Argonaut NCT05480865	Ib	85	Sotorasib + BBP-398 (SHP2 inh)	KRASG12C Solid tumours	Post standard tx	Australia, Spain, Italy, France
NCT04720976	Ib	200	Sotorasib + JAB-3312 (SHP2 inh)	KRASG12C Solid tumours	Several combinations: For KRASG12C + sotorasib	USA
NCT05374538	Ib	140	VIC-1911 (aurora A inh) -/+ sotorasib	KRASG12C NSCLC	Cohort RAS-Is naïve Cohort RAS-Is refractory	USA
NCT05815173	Ib	40	Sotorasib + Ladarixin (IL-8 rec Inh)	KRASG12C NSCLC	Post ICI +/-CT No prior KRAS-I	USA
Neo-kan NCT05472623	II	42	Adagrasib +/- nivolumab	KRASG12C NSCLC	stage IB-IIIA	USA
KRYSTAL-7 NCT04613596	II/III	950	Adagrasib -/+ pembrolizumab Vs CT + pembro	KRASG12C NSCLC	PDL1 <50% Tx naive	Worldwide
NCT05609578	II	90	Adagrasib +/-CT+/- pembrolizumab	KRASG12C NSCLC	Tx naïve PDL1+ PDL1 <50%	USA
RAMP204 NCT05375994	I/II	85	Adagrasib + Avutometinib (VS-6766) (MEK-I)	KRASG12C NSCLC	Prior KRAS-I allowed	USA
KRYSTAL19 NCT05840510	Ib/II	79	Adagrasib + Nab-sirolimus	KRASG12G solid tumours	Part 2 NSCLC	USA
NCT05848843	I	44	Adagrasib + durvalumab	KRASG12G NSCLC and GI	Part 2 NSCLC and CRC Prior RAS-I allowed	USA
NCT05578092	Ib/II	225	MRTX0902 (SOS1 inh) -/+ adagrasib	MAPK alterations NSCLC	Cohort 1 MAPK alterations Cohort 2 KRAS G12C No prior KRAS-	USA I
NCT06024174	I/II	410	Adagrasib BMS-986406 (anti-LILRB2 ab) -/+ cetuximab	G12C solid tumours	Part 2 NSCLC or CRC	USA, Europe, Argentina, Australia, Istrael
KontRASt-01 NCT04699188	I/II	475	JDQ433+ TNO155 (SHP2-I) tislelizumab	KRASG12C solid tumours	Prior RAS-I allowed	Worldwide
KontRASt-03 NCT05358249	Ib/II	346	JDQ433 + trametinib JDQ433 + ribociclib	KRASG12C solid tumours or NSCLC/CRC	Part 2 NSCLC or CRC Post CT and ICI	USA, Europe, Korea, Singapore

			JDQ433 + cetuximab			
KontRASt-04<br>JDQ433C12301<br>NCT TBD	III	380	JDQ433+TNO155<br>Vs<br>Pembro +CT	KRASG12C NSCLC	Tx naive<br>PDL1 three stratification<br>PDL1<1, 1-49%; >50%	Worldwide
NCT04956640	I	400	LY3537982 in several combinations	KRASG12C solid tumours	Post CT +ICI<br>In the cohorts plus pembro or CT tx naïve NSCLC	Worldwide
NCT04973163	I	29	BI 1823911 +<br>BI 1701963<br>(SOS1 inh)<br>Midazolam<br>(PK analysis)	KRASG12C solid tumours	Prior TKIs allowed	USA, Europa
NCT05288205	I/II	124	Glecirasib<br>(JAB-21822) +<br>JAB-3312<br>(SHP2 inhibitor)	solid tumours	Post standard care<br>KRASG12C+ preferred	China
NCT05379946	I/II	92	D-1553 +<br>IN10018<br>(FAK inh)	KRAS G12C solid tumours	-	China
MK-1084-001<br>NCT05067283	I	450	MK-1084 +<br>Several combinations<br>Pembrolizumab<br>Cetuximab<br>CT according to tumour	KRAS G12C solid tumours	Arm2 (pembro)<br>tx naïve KRASG12C /PDL1 + NSCLC<br>Arm 4 (pembro +CT)<br>tx naïve KRASG12C nonsq NSCLC	Worldwide

Abbreviations: Pt N = patient number; vs = versus; NSCLC = non-small-cell lung cancer; KRAS-I(s) = KRAS inhibitors; Tx = treatment; inh = inhibitory; CRC = colon rectal cancer; nonsq = Non squamous; CT = chemotherapy; ICI = immune-checkpoint inhibitor; PD-L1 = Programmed Cell Death Ligand 1; CRT = chemo-radiotherapy; BM = brain metastases; MVASI = bevacizumab biosimilar; TKI = tyrosine kinase inhibitor; MAPK = Mitogen-activated protein kinase.

Table S13. ICI-TKI based combinations in BRAF mutated NSCLC patients.

Trial	Phase	Pt N	Drugs	Disease	Key inclusion criteria	Location
NCT03600701	Ib/II	48	Atezolizumab + cobimetinib	NSCLC	Cohort 1 KRAS+<br>Cohort 2 No KRAS+ refractory to ICI	USA
BFAST<br>NCT03178552	II/III	1000	Vemurafenib<br>Cobimetinib<br>atezolizumab	cfDNA BRAFV600E	A: ALK alectinib<br>B RET alectinib<br>C bTMB atezolizumab vs CT<br>D ROS1 entrectinib<br>E BRAFV600E<br>F EGFREx20ins<br>CT + beva + atezo<br>G KRASG12C<br>GDC-6036 or TXT	Worldwide
IM-BATTLE-2<br>NCT03225664	Ib/II	37	Pembrolizumab + trametinib	NSCLC w/wout EGFR/ALK/ROS1	Refractory to ICI and TKI if gene-addicted NSCLC	USA
NCT05988697	II	36	Aspirin + dabrafenib+ trametinib	BRAFV600E<br>tx naive	No prior tx including BRAF/MEK-I	China
NCT05641493	Ib/II	49	HLX208+<br>Serplulimab<br>(HLX10)<br>Anti-PD-1	BRAFV600E<br>Solid tumours and NSCLC (ph II)	No prior BRAF/MEK-I	China
NCT04777175	Obs	186	ICI	KRAS, BRAF, HER2, MET, ALK, RET	Retrospective study	China

Abbreviations: Pt N = patient number; obs.= observational; NSCLC = non-small-cell lung cancer; KRAS = Ki-ras2 Kirsten rat sarcoma viral oncogene homolog; BRAF = v-raf murine sarcoma viral oncogene homolog B1 KRAS-I(s) = KRAS inhibitors; Tx = treatment; cfDNA circulating free DNA; ICI = immunecheckpoint inhibitor; w = with; wout = without; Tx = treatment naïve; atezo = atezolizumab.

Table S14. Ongoing clinical trials of TKI-based combination in MET dysregulated NSCLC patients.

Trial	Phase	Pt N	Drugs	Disease	Key inclusion criteria	Location
METalmark NCT05488314	Ib/II	161	Capmatinib plus amivantamab	MET+ NSCLC	Cohort 1A tx naïve METex14 NSCLC<br>Cohort 1B pretreated METex14 NSCLC<br>Cohort 1C pretreated MET AMP NSCLC	USA, Turkey, Korea
NCT05435846	Ib	33	Capmatinib plus trametinib	METex14 NSCLC	METex14 on lx or tissue<br>Refractory to prior MET-I<br>Note MET-I is not mandatory to be the last line of treatment	USA
POTENT NCT05782361	Ib	38	Tepotinib plus pembrolizumab	NSCLC with or without METex14	METex14 on lx or tissue<br>Part A Tx Naïve or ICI refractory<br>Part B tx naïve METex14	UK
SOUND NCT05374603	II	60	Savolitinib plus durvalumab	MET+ NSCLC	MET OE, MET AMP or METex14<br>No prior MET-I or ICI	China
NCT05777278	I/II	29	Savolitinib plus docetaxel	MET OE NSCLC	Second line setting<br>MET OE<br>(3+ in ≥50% of TC)	China

Abbreviations = pt N = patient number; Lx = liquid biopsy; MET+ = MET dysregulated; MET OE = MET overexpression; METex14 = MET exon 14 skipping mutations; MET AMP = MET amplification; TC = tumor cells; Tx Naïve= treatment naïve; ICI = immune checkpoint inhibitors; MET-I(s) = MET inhibitor(s); NSCLC = Non-small-cell lung cancer.

Table S15. Ongoing clinical trials of TKI-based in ALK+ or ROS1+ NSCLC patients.

Trial	Phase	Pt N	Drugs	Disease	Key inclusion Criteria	Location
**ALK-I based combo**						
NCT04356118	IV	30	RH endostatin+ intratecal MTX + ALKI	NSCLC	LMC+	China
MASTER ALK NCT05200481	RPII	110	Brigatinib +/- CT	ALK+ NSCLC	Tx naive	France
NCT05491811	II	77	Ensartinib + bevacizumab	ALK+ NSCLC	Tx naïve<br>p53+	China
NCT03202940	Ib/II	31	Alectinib + cobimetinib	ALK+ NSCLC	Post-alectinib	USA
**Other Combo**						
HARMONIC NCT05456256	II	90	LP-300 + CT	Gene-addicted NSCLC	Post standard tx	USA
NCT05266846	II	30	Pembrolizumab+ bevacizumab + CT	ALK+ NSCLC	Post alectinib	China
NCT03991403	RPIII	228	CT + bevacizumab + atezolizumab	EGFR+ or ALK+ NSCLC	Post TKIs	Korea<br>Not recruiting
GFPC 06-2018 NCT04042558	II	149	CT + atezolizumab+/- bevacizumab	EGFR+ or ALK+ ROS1+ NSCLC	Post TKIs	France
NCT04425135	II	59	CT+ camrelizumab+ apatinib	ALK+ NSCLC	Post ALK-Is	China
NCT04989322	II	46	Pembrolizumab + Lenvatinib + CT	EGFR+ or ALK+ ROS1+ NSCLC	Post TKis	Hong Kong
NCT05296278	II	80	IBI-322 + Lenvatinib + CT	ALK+ NSCLC	Post alectinib	China
NCT04777084	Ib	100	IBI-318+ Lenvatinib	NSCLC with or without EGFR/ALK	Post ICI<br>Post TKis	China
NCT05681780	I/II	20	CD40L-Augmented TIL	EGFR, ALK, ROS1 or HER2 NSCLC	Post TKIs or standard tx	USA
PIKACHU	Obs.	100	Anti-PD1 plus CT	ALK+ ROS1+	Post TKis	China

NCT04322890				NSCLC		
NCT05195619	I	16	DC vaccines	NSCLC	Non-gene addicted NSCLC Or EGFR/ALK or ROS-1 post TKI	Switzerland
SNK_ASTER NCT04872634	I/II	24	SNK01 NK + CT + /-cetuximab	EGFR+ or ALK+ ROS1+ NSCLC	Post-TKIs	Korea
NCT04880863	II	35	NAP+ Docetaxel+ Obinutuzumab	NSCLC	NSCLC post ICI Gene addicted NSCLC post TKIs	USA Not recruiting
NCT03645928	II	178	Autologous TILs	Solid tumours	NSCLC post ICI Gene addicted NSCLC post TKIs	USA, Europe

Abbreviations: LMC = leptomeningeal carcinomatosis; ALK = Anaplastic Lymphoma Kinase; EGFR = Epidermal Growth Factor Receptor; ROS1 = c-ros oncogene 1; ALK-I(s) = ALK inhibitor(s); TKIs = tyrosine kinase inhibitor(s); MTX = methotrexate; CT = chemotherapy; PD1 = programmated death-1; DC vaccine = dendritic cell vaccine; TILs = tumor-infiltrating lymphocytes ICI = immune check-point inhibitor; Tx = treatment; NSCLC = Non-small-cell-lung cancer.

Table S16. Ongoing combinations in RET+ NSCLC.

Trial	Phase	Pt N	Drugs	Disease	Key inclusion Criteria	Location
LungMAP NCT05364645	II	74	Selpercatinib + CT	RET+ NSCLC	Progressing on RET-I as the most recent tx line	USA
NCT05845671	I/II	12	Amivantamab + TKis	Gene addicted NSCLC	ALK+, ROS1+, or RET+ Post at least one TKI	USA
**Without TKI**						
POSEIDON NCT04322591	Obs	70	CT vs CT+ ICI	RET+ NSCLC	Tx naïve RET fusion+	China

Abbreviations Pt N = patient number; TKIs = tyrosine kinase inhibitors; NSCLC = Non-Small-Cell lung cancer; RET = rearranged during transfection; RET-I = RET inhibitors; tx = treatment; CT chemotherapy; ICI = immunocheckpoint.

References

1. Zhou C, Ramalingam SS, Kim TM, et al: Treatment Outcomes and Safety of Mobocertinib in Platinum-Pretreated Patients With EGFR Exon 20 Insertion-Positive Metastatic Non-Small Cell Lung Cancer: A Phase 1/2 Open-label Nonrandomized Clinical Trial. JAMA Oncol 7:e214761, 2021
2. Le X, Goldman JW, Clarke JM, et al: Poziotinib shows activity and durability of responses in subgroups of previously treated EGFR exon 20 NSCLC patients. Journal of Clinical Oncology 38:9514-9514, 2020
3. Yu HA, Tan DS-W, Smit EF, et al: Phase (Ph) 1/2a study of CLN-081 in patients (pts) with NSCLC with EGFR exon 20 insertion mutations (Ins20). Journal of Clinical Oncology 40:9007-9007, 2022
4. Wang M, Yang JC, Mitchell PL, et al: Sunvozertinib, a Selective EGFR Inhibitor for Previously Treated Non-Small Cell Lung Cancer with EGFR Exon 20 Insertion Mutations. Cancer Discov 12:1676-1689, 2022
5. Han B, Zhou C, Wu L, et al: 1210P Preclinical and preliminary clinical investigations of furmonertinib in NSCLC with EGFR exon 20 insertions (20ins). Annals of Oncology 32:S964, 2021
6. Robichaux JP, Elamin YY, Tan Z, et al: Mechanisms and clinical activity of an EGFR and HER2 exon 20-selective kinase inhibitor in non-small cell lung cancer. Nat Med 24:638-646, 2018
7. Le X, Cornelissen R, Garassino M, et al: Poziotinib in Non-Small-Cell Lung Cancer Harboring HER2 Exon 20 Insertion Mutations After Prior Therapies: ZENITH20-2 Trial. J Clin Oncol 40:710-718, 2022

8. Liu SV, Villaruz LC, Lee VHF, et al: LBA61 First analysis of RAIN-701: Study of tarloxotinib in patients with non-small cell lung cancer (NSCLC) EGFR Exon 20 insertion, HER2-activating mutations & other solid tumours with NRG1/ERBB gene fusions. Annals of Oncology 31:S1189, 2020
9. Zhou C, Li X, Wang Q, et al: Pyrotinib in HER2-Mutant Advanced Lung Adenocarcinoma After Platinum-Based Chemotherapy: A Multicenter, Open-Label, Single-Arm, Phase II Study. J Clin Oncol 38:2753-2761, 2020
10. Oguchi K, Araki H, Tsuji S, et al: TAS2940, a novel brain-penetrable pan-ERBB inhibitor, for tumors with HER2 and EGFR aberrations. Cancer Sci 114:654-664, 2023
